# Life Stage-Dependent Toxicity and Interactions of Scrubber-Related Metal Mixtures in Marine Zooplankton

**DOI:** 10.3390/toxics14060530

**Published:** 2026-06-19

**Authors:** Esther Bautista-Chamizo, María Cabrera-Bayarri, Enrique Nebot, Javier Moreno-Andrés

**Affiliations:** 1 Department of Environmental Technologies, Marine Research Institute (INMAR), Faculty of Marine and Environmental Sciences, University of Cádiz, 11510 Puerto Real, Spain; maria.cabrerabayarri@alum.uca.es (M.C.-B.); enrique.nebot@uca.es (E.N.); javier.moreno@uca.es (J.M.-A.); 2 Microbiology and Proteomics Laboratory, Department of Biomedicine, Biotechnology and Public Health, Institute for Viticulture and Agri-Food Research (IVAGRO), Faculty of Marine and Environmental Sciences, University of Cádiz, 11510 Puerto Real, Spain

**Keywords:** Cumulative Toxic Unit, exhaust gas cleaning system (EGCS), LC_50_, marine pollution, mixture toxicity, shipping, scrubber washwater

## Abstract

The adoption of exhaust gas cleaning systems (scrubbers) in maritime transport generates a complex metal-laden washwater that may pose a noteworthy threat to marine ecosystems. This study assessed the acute toxic effects (LC_50_, 48 h) of four prevalent metals detected in scrubber washwater—vanadium (V), iron (Fe), nickel (Ni), and zinc (Zn)—both individually and as a realistic mixture. For this purpose, multiple life stages of *Artemia franciscana* (nauplii, juveniles, and adults) and the rotifer *Brachionus plicatilis* have been tested under laboratory conditions. All metals induced concentration-dependent toxicity, but sensitivities varied through life stages and species tested. The sensitivity to contaminants generally decreased as the organism’s developmental stage progressed. Consequently, three different orders of toxicity can be detected. The order of metal toxicity (from highest to lowest toxicity, based on 48 h LC_50_ values) was V > Fe > Ni > Zn for nauplii; V > Zn > Fe > Ni for juveniles and adults; and Fe > V > Zn > Ni for *B. plicatilis*. The Cumulative Toxic Unit (CTU) approach was utilized to compare the predicted additive effect with observed mixture toxicity. This analysis revealed a complex, life stage-dependent interaction; while antagonism dominated in nauplii (suggesting chemical mitigation), juveniles and adults of *A. franciscana* and the rotifer (*B. plicatilis*) exhibited significant synergism, amplifying the total toxicity beyond prediction. This study demonstrates that early life stages and small zooplankton are the most sensitive bioindicators of scrubber-related metal contamination, highlighting the potential ecological risk posed by metal-rich, acidic scrubber discharges that may enhance metal bioavailability and toxicity in marine environments.

## 1. Introduction

Maritime transport is fundamental to the global economy, moving nearly 90% of international merchandise trade and growing by 2.2%, exceeding the 2013–2023 average (1.8%) [1]. According to the MARPOL Convention [2], this activity generates significant environmental impacts, including the discharge of harmful liquid substances, wastewater, and the release of pollutants into the atmosphere. Over 90% of the active fleet by tonnage still runs on conventional fuels [1]. The combustion of heavy fuels, such as fuel oil, releases sulfur oxides (SOx), nitrogen oxides (NOx), particulate matter, and black carbon, posing risks to both environmental integrity and human health, particularly in coastal areas [3].

To mitigate these emissions, the International Maritime Organization (IMO), under MARPOL Annex VI, established a global limit of 0.5% sulfur content in marine fuels in 2020. This limit is further reduced to a maximum of 0.10% by mass in Emission Control Areas (ECAs) [4]. One of the most widely adopted compliance strategies by shipping companies is the installation of Exhaust Gas Cleaning Systems (scrubbers) [5]. This technology allows continued use of traditional fuels while achieving required reductions in atmospheric emissions of sulfur oxides (SOx) and particulate matter [6].

Scrubbers operate by washing exhaust gases with a liquid solution (commonly seawater). The flow of this acidic, high-temperature water through complex piping and the scrubbing mechanism itself drives a high concentration of pollutants into the washwater discharge [7]. Open-loop scrubber systems are particularly problematic as they discharge washwater directly into the sea without treatment. The effluent is characterized by low pH (pH < 4), high temperatures (T > 30 °C), a high content of polycyclic aromatic hydrocarbons (PAHs), and elevated levels of heavy metals, among which vanadium (V) is the most abundant, followed by nickel (Ni), zinc (Zn), iron (Fe), and copper (Cu). Consequently, in areas with limited water renewal or high traffic, these discharges could elevate dissolved metal concentrations above recommended ecological limits [8]. The potential ecotoxicological risks associated with EGCS discharges have already prompted several European nations to implement regional restrictions or bans on ports and territorial waters, highlighting the urgent need for global standardized discharge guidelines [5].

The toxicity of these effluents cannot be predicted by assuming simple additivity of the effects of individual components, as synergistic or antagonistic interactions frequently alter the overall ecological risk. Recent experimental evidence underscores the need for comprehensive ecotoxicological assessments, as adverse effects have been documented across multiple trophic levels even at environmentally relevant dilutions of less than 1%, indicating that the discharge of scrubber effluents, particularly from open-loop systems, poses a significant environmental hazard [9]. These impacts include acute toxicity in mesozooplankton and copepods, affecting survival and grazing rates [10,11], as well as significant shifts in phytoplankton and microplankton community structures [12,13]. Furthermore, the high sensitivity observed in the embryonic and larval stages of marine invertebrates suggests potential for widespread disruption of marine food webs and biogeochemical cycles [14,15,16].

The heavy metals in scrubber washwater could pose a significant risk due to their complex mixture, high environmental persistence, and capacity for bioaccumulation and biomagnification within the food chain [17]. Metal contamination rarely occurs in isolation, yet current environmental risk assessments and quality benchmarks still focus primarily on single-metal impacts [18]. This methodology assumes the total risk is equivalent to the most toxic element present, overlooking the reality that metals can combine to produce additive or synergistic effects [19].

Unlike organic pollutants, metals are not subject to biodegradation and therefore persist in the environment, where they may undergo redistribution, transformation, or immobilization depending on prevailing physicochemical conditions [20,21]. In the specific context of scrubber effluents, the characteristically low pH, substantially lower than ambient seawater, acts as a critical driver of metal bioavailability [22]. Consequently, the simultaneous release of multiple metals in these acidic streams may give rise to complex mixture interactions, such as synergistic or antagonistic toxic effects [23]. Furthermore, heavy metals undergo bioaccumulation, a process in which their internal concentrations progressively increase within aquatic organisms due to chronic exposure [24].

Given the pronounced toxicity and environmental persistence of heavy metals, rigorous surveillance of their release and subsequent bioaccumulation is essential. Such monitoring is critical for elucidating their toxicological effects and forecasting biological responses within affected ecosystems [25]. While most toxicological research focuses on isolated metal species, aquatic organisms are typically exposed to complex mixtures in their natural habitats. Mixture toxicity may be strictly additive, or it may deviate from the sum of its individual components due to synergistic or antagonistic interactions [26].

Based on these considerations, it was hypothesized that the complex metal mixture characteristic of scrubber washwater would cause synergistic toxic effects rather than strict additivity, and that these interactive effects would vary significantly depending on the species and developmental life stage exposed. To test this, the primary objective of this study was to conduct a comprehensive assessment of the acute toxic effects of vanadium (V), zinc (Zn), nickel (Ni), and iron (Fe), both individually and as a representative mixture. These effects were systematically evaluated across different life stages (nauplii, juveniles, and adults) of the brine shrimp *Artemia franciscana* and the rotifer *Brachionus plicatilis*. For *B. plicatilis*, this 48-h assay represents a short-term acute exposure that encompasses a substantial portion of the rotifer’s total lifespan. Using 48 h median lethal concentrations (LC_50_), the Concentration Addition (CA) model was applied to quantify whether the metal interactions resulted in synergism, antagonism, or additive effects.

## 2. Materials and Methods

### 2.1. Reagents and Metal Stock Solutions

The metals studied were V, Zn, Ni, and Fe, as well as a mixture of these four elements at different proportions, simulating real scrubber washwater metal concentration. High-purity metal salts were used in the experiments: zinc sulfate heptahydrate (ZnSO_4_ · 7H_2_O), nickel sulfate hexahydrate (NiSO_4_ · 6H_2_O), and ferrous sulfate heptahydrate (FeSO_4_ · 7H_2_O). For vanadium, sodium metavanadate (NaVO_3_) was used since this element is predominantly found in seawater in its pentavalent oxidation state, mainly as vanadate species, as described in the literature [27]. All salts were purchased through Sigma-Aldrich (St. Louis, MO, USA).

A concentrated stock solution was prepared for each metal. To verify the desired stock’s metal concentration, 10 mL samples from each stock solution were taken immediately after preparation and acidified with Suprapur nitric acid (pH < 2) for preservation. These samples were stored in 15 mL Falcon tubes, refrigerated at 4 °C in the dark [28] for subsequent analysis. Chemical analyses were performed using a mass spectrometer (ICP-MS/Thermo Elemental Series-X, Winsford, UK) at the Spectroscopy Division of the “Central Science and Technology Services” of the University of Cádiz (see Table S1 in Supplementary Material).

The selection of metal concentrations was based on values reported in previous studies [7,22,29] and complemented by the authors’ analysis of real scrubber washwater samples collected from a ship (Table 1). Exposure concentrations for individual metals ranged from 0.1 to 200 mg/L for *A. franciscana* (0.1, 1, 5, 10, 20, 30, 40, 100, 150, and 200 mg/L). However, for *B. plicatilis*, the upper concentration limit was extended up to 500 mg/L (0.1, 1, 5, 10, 20, 30, 40, 100, 250, and 500 mg/L) for Zn, Ni and Fe, while V was tested at 0.1, 1, 5, 10, 20, 30, 40, 50, 75, and 100 mg/L. In both cases, the mixture concentrations ranged from 0.1 to 100 mg/L (0.1, 1, 5, 10, 15, 20, 30, 50, 75, and 100 mg/L). These wide exposure ranges were determined based on preliminary range-finding tests conducted in the laboratory to ensure sufficient mortality data for accurate LC_50_ calculation and to evaluate mixture interactions. Furthermore, these experimental ranges were strategically selected to encompass environmentally realistic concentrations reported for actual scrubber washwater discharges, ensuring that the ecotoxicological thresholds determined in this study are directly relevant to real-world pollution scenarios, while simultaneously allowing for the assessment of a worst-case scenario (e.g., undiluted effluents in the immediate vicinity of the discharge plume or within high-traffic, semi-enclosed ports).

The metal mixture was designed according to the research group’s results, as follows: 3% Zn, 15% Ni, 37% V, and 45% Fe, testing progressively increasing concentrations ranging from 0.1 to 100 mg/L (0.1, 1, 5, 10, 15, 20, 30, 50, 75, and 100 mg/L).

### 2.2. Toxicity Tests

Two marine species, widely used in toxicity tests [30,31], were employed in this study: *Artemia franciscana*, widely recognized as a model organism [32], and *Brachionus plicatilis* [33]. For *A. franciscana*, organisms at three developmental stages were used: nauplii (newly hatched individuals), juveniles (15 days old), and adults (26 days old). In the case of *B. plicatilis*, under standard laboratory conditions, this species exhibits a rapid development and a total lifespan ranging from approximately 3 to 24 days [34,35]. Organisms were provided by the Marine Culture Service of the Faculty of Marine and Environmental Sciences at the University of Cádiz (UCA).

Toxicity tests were developed with filtered seawater (Millipore 22 μm) collected in the Bay of Cádiz, in sterilized 24-well plates for nauplii and rotifers (2 mL capacity per well) and in 12-well plates for juvenile and adult brine shrimp (5 mL capacity per well). For the experiments involving *A. franciscana*, groups of 20 to 30 nauplii were transferred to each experimental well, while 15 to 20 individuals were introduced per well for the juvenile and adult bioassays. For *B. plicatilis*, around 50 rotifers were added per well. Six replicates were prepared for each metal concentration.

Each well was filled with 20 μL of microalgae (*Tetraselmis chuii*) as food, along with the required amount of metal stock solution and filtered seawater to a volume of 2 or 5 mL, depending on the plate type. The experiments lasted 48 h, with live and dead organisms counted to assess mortality. All microplates were placed inside a chamber with controlled lighting (16:8-h light/dark cycle), humidity (55%), and temperature (24 °C).

For *A. franciscana*, individual metals (V, Zn, Ni, and Fe) were tested at ten progressively increasing nominal concentrations ranging from 0.1 to 200 mg/L. Similarly, *B. plicatilis* was exposed to ten concentrations ranging from 0.1 to 100 mg/L (V) or 500 mg/L (Zn, Ni, and Fe). In both organisms, the mixture toxicity was evaluated between 0.1 and 100 mg/L.

In the case of *B. plicatilis*, it was necessary to filter the Fe stock to remove the insoluble particles of this metal, since the formation of precipitated particles caused immobilization and subsequent death of the rotifers due to their small size, as they became trapped under these particles. Thus, analytical verifications confirmed that the filtered Fe concentration in the rotifer testing media was 20% lower than the nominal value.

### 2.3. Data Processing and Statistical Analysis

To analyze the results of the experiment, a Microsoft Excel spreadsheet (Microsoft Corp., Redmond, WA, USA) was used to record raw data on live and dead organisms at 48 h. All organisms that had completely lost movement were counted as dead. The values obtained for each replicate were recorded based on the organism, the developmental stage (in the case of *A. franciscana*), the metal tested, and its concentration.

To determine and evaluate significant differences between all treatments and the control (*p* < 0.05), confidence intervals (95%) were calculated for each parameter and for each species. Statistical analysis was performed using SPSS 15.0 software (SPSS Inc., Chicago, IL, USA).

The median lethal concentration (LC_50_) was estimated for each metal, defined as the concentration of a toxic substance expected to cause the death of 50% of the organisms exposed to it in the given period. A nonlinear regression model was used for calculation, following the methodology described by Hampel et al. [36].

The model of Hampel et al. [36] was fitted to the data, yielding an R^2^ > 0.9 in all cases, which validates the high quality of the fit (see Figure S1 in Supplementary Material). The key parameter, T (LC_50_), proved to be statistically significant (*p*-value < 0.05), confirming the model’s predictive validity and robustness (see Table S1 in Supplementary Material).

The fit was performed using SigmaPlot version 10.0 (Systat Software Inc., San Jose, CA, USA), obtaining the LC_50_ values along with the coefficient of determination (R^2^) for each fit and its corresponding statistical significance (*p*) value. All graphical representations were generated using GraphPad Prism version 9.0 software (GraphPad Software, Boston, MA, USA).

The Concentration Addition (CA) model was employed to predict the acute toxicity of the mixture, assuming that components with the same mode of action act in an additive manner [37,38]. Under this framework, individual toxic units (TU_i_) were calculated for each measured metal (i) in the mixture according to Sprague [39], TU_i_ = (C_i_/LC_50i_), where Ci is the average measured concentration of each metal. The predicted cumulative toxicity of the mixture (CTU_predicted_) was then calculated as the sum of the TU_i_ values of each metal (CTU_predicted_ = ∑ TU_i_) [40].

The actual observed acute toxicity of the mixture was expressed as the Observed Cumulative Toxic Unit (CTU_observed_), derived from the 48 h LC_50_ of the mixture (CTU_observed_ = C_mix_/LC_50mix_). For this calculation, the total metal concentration of the synthetic mixture was set at 15.00 mg/L, representing the cumulative sum of the average concentrations of V (5.55 mg/L), Fe (6.75 mg/L), Ni (2.25 mg/L), and Zn (0.45 mg/L) found in scrubber washwater according to the research group’s results (Table 1).

Finally, the Toxicity Ratio (TR = CTU_observed_/CTU_predicted_) was calculated to quantify the magnitude of synergistic or antagonistic deviation from the CA model [41]. According to the thresholds established by Norwood et al. [23], a TR = 1.0 indicates additivity (observed toxicity equals predicted toxicity); a TR > 1.0 indicates synergism (observed toxicity is higher than predicted); and a TR < 1.0 indicates antagonism (observed toxicity is lower than predicted).

## 3. Results

### 3.1. Mortality Rate and LC_50_ Evaluation

#### 3.1.1. *Artemia franciscana*

**Figure 1:**
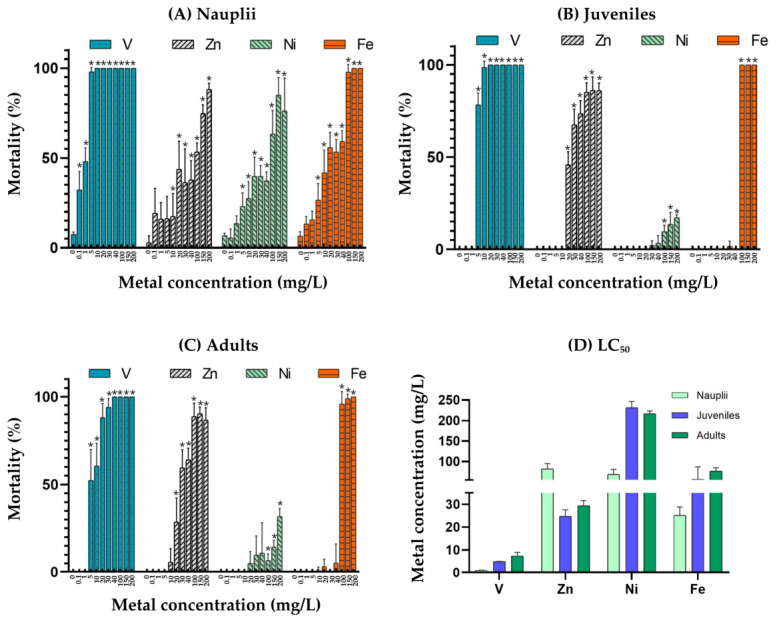
Mortality percentage of *Artemia franciscana* after 48 h exposure to different concentrations of heavy metals (V, Zn, Ni, Fe) across developmental stages: (**A**) Nauplii, (**B**) Juveniles, (**C**) Adults. (**D**) summarizes the LC_50_ values derived using the model of Hampel et al. [36]. Metal concentrations tested were 0, 0.1, 1, 5, 10, 20, 30, 40, 100, 150, and 200 mg/L. Data represent mean ± S.D. (*n* = 6 replicates). * Indicates a statistically significant difference compared to the control (*p* < 0.05).

The mortality rate in nauplii, juveniles, and adults of *A. franciscana* was assessed after 48 h of exposure to increasing concentrations of V, Fe, Ni, and Zn, tested individually (Figure 1). LC_50_ experimental and modeled data are presented in Figure S1, and model parameters in Table S1.

*A. franciscana* exhibited a clear dose–response relationship, with mortality increasing progressively as the metal concentration increased. Compared to juveniles and adults (Figure 1B,C), nauplii (Figure 1A) showed a greater overall sensitivity to heavy metals. Vanadium was found to be the most toxic element, being particularly notable in nauplii, where concentrations of just 5 mg/L caused mortality rates exceeding 95% (Figure 1A). This lethal effect required higher concentrations in juveniles (LC_50_ of 4.95 mg/L ± 0.04) and adults (LC_50_ of 7.39 mg/L ± 1.59) (Figure 1D).

Zinc appeared to be the least toxic metal in nauplii (LC_50_ = 98.3 ± 3.32 mg/L) but exhibited intermediate toxicity in juveniles (LC_50_ = 24.71 ± 2.78 mg/L) and adults (LC_50_ = 29.37 ± 2.23 mg/L) (Figure 1D). Nonetheless, it did not induce total mortality at any stage at the highest concentration tested.

However, for *A. franciscana* adults and juveniles, Ni was the least toxic metal evaluated, with an LC_50_ above 200 mg/L, which was significantly lower than that of nauplii (LC_50_ = 68.59 ± 12.28 mg/L) (Figure 1D).

Iron toxicity was more gradual in nauplii, with significant effects beginning at 5 mg/L, exceeding 50% mortality at 20 mg/L, and reaching 100% at concentrations above 100 mg/L. The LC_50_ values (Figure 1D) indicate that *A. franciscana* sensitivity varies significantly by both metal type and life stage. For all metals tested, a clear trend of increasing tolerance was observed as development progressed from nauplius to adults, except for Zn. Specifically, two different orders can be detected: for nauplius V > Fe > Ni > Zn, and for juveniles and adults V > Zn > Fe > Ni.

#### 3.1.2. *Brachionus plicatilis*

**Figure 2:**
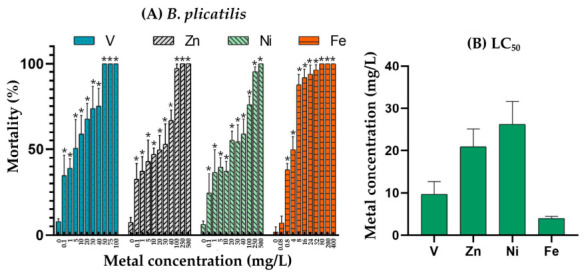
(**A**) Mortality percentage of *Brachionus plicatilis* after 48 h exposure to different concentrations of heavy metals (V, Zn, Ni, Fe); (**B**) LC_50_ values after 48 h exposure calculated using the model of Hampel et al. [36]. Tested concentrations ranged from 0.1 to 500 mg/L (0.1, 1, 5, 10, 20, 30, 40, 100, 250, and 500 mg/L) for Zn, Ni, and Fe, and from 0.1 to 100 mg/L (0.1, 1, 5, 10, 20, 30, 40, 50, 75, and 100 mg/L) for V. Iron values are expressed as nominal concentrations for comparative purposes. Data represent mean ± SD (*n* = 6 replicates). * Indicates a statistically significant difference compared to the control (*p* < 0.05).

The mortality results obtained in *B. plicatilis* after 48 h of exposure to heavy metals are presented in Figure 2. LC_50_ experimental and modeled data are presented in Figure S1, and model parameters in Table S1.

Vanadium was the most toxic agent after Fe in *B. plicatilis*, causing statistically significant mortality from 0.1 mg/L (LC_50_ = 9.68 ± 2.95 mg/L, Figure 2B). Zinc generated an appreciable, yet gradual toxic effect, inducing significant mortality from 0.1 mg/L (LC_50_ = 20.93 ± 4.23 mg/L, Figure 2B). The Zn toxicity profile was relatively consistent between species. The results indicate a higher sensitivity of *B. plicatilis* to this metal compared to *A. franciscana*. Specifically, rotifers reached 100% lethality at 250 mg/L (Figure 2A), whereas *A. franciscana* did not reach this level at 200 mg/L (Figure 1).

Nickel was the least toxic metal evaluated, generating a gradual effect and requiring 500 mg/L to achieve maximum lethal effect in *B. plicatilis* (LC_50_ = 26.25 ± 5.43 mg/L, Figure 2B).

Iron exhibited a particularly aggressive response on *B. plicatilis*, with significant effects at 1 mg/L (LC_50_ = 3.95 ± 0.46 mg/L, Figure 2B), and total lethality at 100 mg/L (Figure 2A). The intensity of Fe toxicity in rotifers greatly exceeded the response of *A. franciscana* nauplii, juveniles, and adults.

### 3.2. Metal Mixture: Mortality, LC_50,_ and Cumulative Toxic Unit

**Figure 3:**
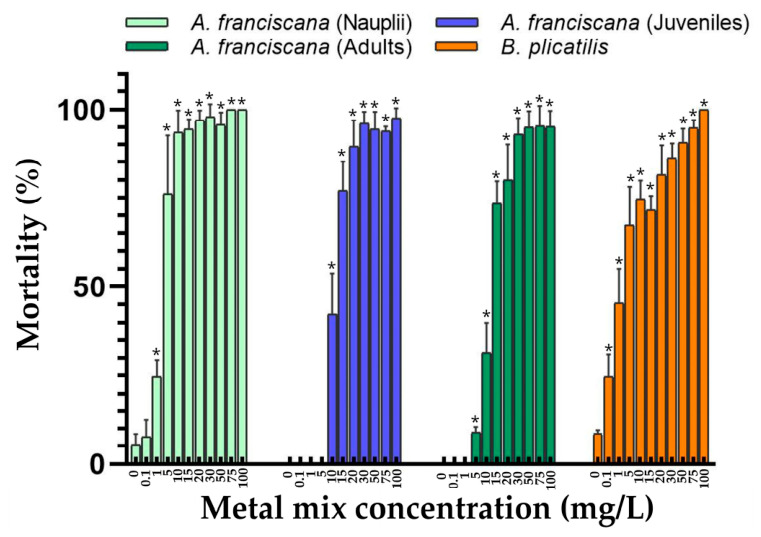
Mortality of *Artemia franciscana* (nauplii, juveniles, and adults) and *B. plicatilis* after 48 h exposure to different concentrations of mixed heavy metals (V, Zn, Ni, and Fe). Metal concentrations tested were 0.1, 1, 5, 10, 15, 20, 30, 50, 75, and 100 mg/L. Data represent mean ± S.D. (*n* = 6 replicates). * Indicates a statistically significant difference compared to the control (*p* < 0.05).

**Figure 4:**
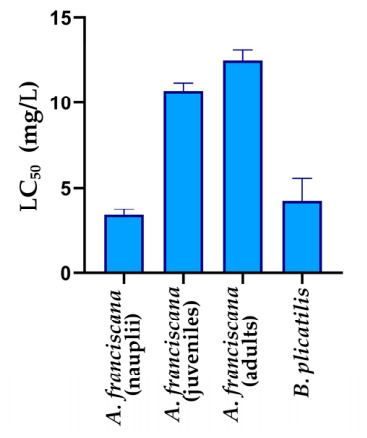
LC_50_ of *Artemia franciscana* (nauplii, juveniles, and adults) and *B. plicatilis* after 48 h exposure to different concentrations of mixed heavy metals (V, Zn, Ni, and Fe). Metal mix concentrations tested were 0.1, 1, 5, 10, 15, 20, 30, 50, 75, and 100 mg/L. These values were obtained using the model of Hampel et al. [36]. Data represent mean ± S.D. (*n* = 6 replicates).

Figure 3 illustrates a clear dose-dependent response to the metal mixture across all tested organisms. *B. plicatilis* showed the greatest vulnerability, with effects detected as early as 0.1 mg/L (LC_50_ = 4.29 ± 1.27 mg/L, Figure 4). In *A. franciscana*, nauplii were the most sensitive stage, with mortality starting at 1 mg/L (Figure 3) (LC_50_ = 3.43 ± 0.33 mg/L, Figure 4). Juveniles exhibited intermediate sensitivity (LC_50_ = 10.70 ± 0.47 mg/L, Figure 4), and the tolerance of adults was comparable to that of juveniles (LC_50_ = 12.47 ± 0.62 mg/L, Figure 4), which is a concerning result, since the effluent’s average concentration of metals calculated from the compiled data (Table 1) is approximately 19.9 ± 10.0 mg/L.

The acute toxicity of the synthetic metal mixture (Table 2) demonstrated highly variable interactions according to the life stage of *A. franciscana*. For *A. franciscana* nauplii, the CTU_observed_ (4.37) was lower than the CTU_predicted_ (5.75), resulting in a Toxicity Ratio (TR) of 0.76. According to the model’s thresholds, a TR below 1.0 indicates an antagonistic effect. In contrast, *A. franciscana* juveniles exhibited a slight synergism (TR = 1.11), which became even more pronounced in adults (TR = 1.39). Across all stages of the brine shrimp, the individual toxicity of V was considerably higher than that of the other single metals (see Table S3 in the Supplementary Material). Furthermore, this synergistic effect was maximized in the rotifer *B. plicatilis* (CTU_Observed_ = 3.5 vs. CTU_Predicted_ = 2.06; TR = 1.70), an organism for which Fe was the primary metal contributing to the predicted toxicity.

## 4. Discussion

### 4.1. Toxicity of Individual Metals in Marine Zooplankton

Metals can be classified as essential or non-essential. Essential elements such as Zn, Ni, and Fe are required for biological processes but become toxic when their concentrations exceed physiological thresholds or fall below optimal levels [42]. In contrast, non-essential metals such as V have no known biological function but can mimic essential metals, allowing them to bind to biological ligands and enter organisms through gill or digestive epithelia. This molecular mimicry enables them to bypass regulatory mechanisms and interfere with metal-dependent cellular processes [43]. Once internalized, metals exert toxicity through multiple mechanisms, including inhibition of enzymatic activity, generation of reactive oxygen species (ROS), disruption of ionic homeostasis, and formation of DNA or protein adducts [44].

Vanadium was the most toxic metal across all stages of *A. franciscana*, highlighting its relevance as a priority marine pollutant. This finding aligns with previous studies identifying V as a hazardous emerging contaminant [45], despite the currently limited ecotoxicological data available on aquatic organisms. Reported effects include strong enzymatic inhibition during critical developmental stages in marine invertebrates [46], as well as adverse responses at relatively low concentrations, such as toxicity in *Daphnia magna* at 2.7 mg/L and developmental alterations in *Paracentrotus lividus* embryos at 100 μg/L [47]. These observations confirm that V can disrupt key physiological processes, including ionic transport, enzymatic activity, and cellular homeostasis, even at submillimolar concentrations [45,48]. Mechanistically, its high toxicity is largely attributed to its ability to inhibit Na^+^-K^+^-ATPase activity [49], thereby disrupting ion regulation [45]. This effect has been demonstrated in different taxa, including *Anguilla anguilla* (0.1 to 10 µM as orthovanadate) and *Cordylophora caspia* (1.74 to 7.96 mg V/L as ammonium metavanadate) [50,51]. The higher sensitivity observed in *Artemia* compared to rotifers may be explained by their strong dependence on this enzyme for osmoregulation in hypersaline environments, making them particularly vulnerable to V-induced disruption [52].

Zinc exhibited a life stage-dependent toxicity pattern in *Artemia*, being less toxic to nauplii than to juveniles and adults, likely due to differences in exposure routes. In nauplii, exposure is mainly limited to direct contact with dissolved Zn, as individuals still rely on yolk reserves, whereas juveniles and adults actively ingest microalgae, which can rapidly bioaccumulate Zn because it is an essential element for algal growth [53]. As a result, older stages were subjected to both aqueous and dietary exposure, increasing the internal dose and accelerating lethal effects, in agreement with previous reports showing that trophic transfer of metal-enriched prey can enhance toxicity in *Artemia* and other zooplankton [54]. This interpretation is consistent with the known mechanisms of Zn toxicity in marine organisms, which include direct cellular disruption by Zn^2+^ ions [55], ROS generation and oxidative stress [56], and competition with essential elements such as calcium, thereby altering key metabolic processes [57]. These effects are further intensified by the high assimilation efficiency of Zn through both direct and dietary pathways, reaching 50–99% in marine organisms, and by its potential for biomagnification along the food web [58], although the final toxicity remains strongly dependent on nutrient conditions, species, and ambient Zn concentrations [59]. The high sensitivity observed in *B. plicatilis* is also consistent with previous reports for other Zn forms, such as ZnO nanoparticles, which showed an LC_50_ of 12.43 mg/L at 48 h [60], supporting its use as a sensitive indicator of Zn-related stress regardless of Zn speciation.

Nickel showed moderate toxicity in the present study, consistent with its known biological effects, which include disruption of calcium and magnesium homeostasis, induction of oxidative stress, and impairment of respiratory processes [61,62]. However, its toxicity is highly species-specific and context-dependent [63], as reflected in the wide variability reported in the literature. A meta-analysis covering 40 tropical freshwater species revealed a broad toxicity range (1.4–419,000 μg Ni/L), with invertebrates displaying highly variable sensitivities, from moderate to extreme tolerance (acute EC_50_ values of 460–155,000 μg Ni/L) [64]. Similarly, marine organisms have shown considerable tolerance to Ni, including *A. franciscana* and bivalves, with EC_50_ values ranging from 251.7 to 607.2 mg/L in mussel larvae and up to 891 mg/L in *Mytilus* spp. [65,66]. In this context, the moderate toxicity observed in *B. plicatilis* suggests a relatively higher sensitivity compared to other rotifers, such as *Proales similis*, which exhibits much greater tolerance (LC_50_ > 2000 mg/L) [67], highlighting the substantial variability in Ni tolerance within the Rotifera phylum.

The greater tolerance to Fe observed in *A. franciscana* juveniles and adults (LC_50_ = 56.39 ± 3.35 mg/L and 76.39 ± 8.36 mg/L, respectively) aligns with findings by Migliore and de Nicola Giudici [68], who documented greater Fe tolerance in more advanced stages of the crustacean *Asellus aquaticus*. Although Fe is essential for marine organisms [69], it exhibited a particularly aggressive response in *B. plicatilis*, with significant effects at 1 mg/L (LC_50_ = 3.95 ± 0.46 mg/L), and total lethality at 100 mg/L. The intensity of Fe toxicity in rotifers greatly exceeded the response of *A. franciscana* nauplii, juveniles, and adults. This high sensitivity may be explained by the ability of Fe to induce severe oxidative stress via the Fenton reaction, which generates highly cytotoxic hydroxyl radicals [70]. Previous research found that ROS-triggered oxidative stress could inactivate the enzymatic antioxidant activity in the rotifer *B. plicatilis*, worsening the overproduction of ROS [71]. Unlike Zn or Ni, whose toxicity usually stems from competition for ion channels, Fe has been associated with negative effects on rotifer reproductive processes [72]. This greater susceptibility is likely exacerbated by the formation of ferric precipitates, which can adhere to the rotifer body surface, interfering with vital functions such as feeding and locomotion [73]. This physicochemical stress is a key factor in static bioassays and aligns with observations of respiratory impairment caused by high precipitated Fe in other species [74].

The observed differences in toxicity endpoints suggest an increased capacity for metal regulation and absorption in more advanced stages, likely due to the development of physiological barriers and more efficient ionic regulation [75]. Additionally, the combined effects of species differences, developmental stage, and interspecific variability in metal tolerance highlight the importance of multi-species and multi-stage approaches in ecotoxicological assessments. Similar evidence of pronounced interspecific sensitivity differences has been reported in ecotoxicological studies of scrubber washwater, for instance, where blue mussel (*Mytilus edulis*) larvae were found to be highly vulnerable compared to the robust tolerance displayed by the unicellular algae (*Tetraselmis suecica*) under metal exposure [16].

### 4.2. Toxicity and Interactions of Metal Mixtures

The assessment of metal mixtures is inherently complex due to the essential nature of many metals, which has driven marine organisms to develop sophisticated uptake, storage, and detoxification mechanisms, including metallothionein induction and metal-binding ligands [38,76]. Despite this, studies on metal mixture toxicity have been predominantly conducted in freshwater systems [64], with comparatively few addressing marine organisms, such as *Mytilus edulis* [65], *Paracentrotus lividus* [77], or Antarctic microalgae [78]. Existing marine studies highlight complex and concentration-dependent interactions; for instance, synergism between Cu and Ni has been reported at environmentally relevant levels, while also emphasizing that extrapolation across concentration ranges may lead to inaccurate toxicity predictions [65]. Interactions among metals can occur already at the bioavailability and uptake stages, where competition for transport pathways or ligand binding may enhance or inhibit accumulation, as shown by Cu reducing lead uptake in mussels [79] and Zn-induced metallothionein-like proteins increasing cadmium (Cd) accumulation in oysters [80]. These early interactions ultimately determine downstream toxic effects, leading to synergistic, additive, or antagonistic responses across taxa [81,82]. In agreement with this pattern, results showed an age-dependent shift in mixture interactions within *A. franciscana*, ranging from antagonism in nauplii to slight synergism in juveniles and stronger synergistic effects in adults. The antagonistic response observed in nauplii is consistent with competition among dissolved metals for a limited number of membrane binding and transport sites, which can reduce the net uptake of individual ions and lower overall toxicity [83,84]. During early development, *A. franciscana* nauplii rely on generalized ion transport pathways and intense osmoregulatory activity to sustain rapid growth before the full development of branchial ionoregulatory structures [85,86]. Under these conditions, exposure to a multi-metal mixture (V, Zn, Ni, Fe) may promote strong competition among metals for shared uptake pathways, limiting internal accumulation and favoring antagonistic interactions. Furthermore, the rapid growth rate of nauplii may facilitate somatic growth dilution (SGD), a process where a continuous increase in biomass effectively reduces intracellular metal concentrations [87]. In contrast, the synergistic effects observed in juveniles, adults, and *B. plicatilis* suggest a shift from uptake-limited toxicity to detoxification-limited toxicity. As organisms develop, feeding activity and ion uptake become more stable and efficient, increasing the potential for metal accumulation [86]. Under these conditions, intracellular defense systems such as metallothioneins [88], glutathione-related pathways [89], and antioxidant enzymes (e.g., catalase, CAT, and glutathione-S-transferase, GST) [90] may become progressively saturated during simultaneous exposure to multiple stressors. Once these detoxification pathways are overwhelmed, the accumulated metals can induce cumulative oxidative damage through shared mechanisms involving reactive oxygen species (ROS) production and lipid peroxidation, resulting in synergistic toxicity [91]. The strong synergistic responses observed in *B. plicatilis* may also reflect the comparatively limited detoxification capacity and short life cycle of rotifers, which make them especially sensitive to multi-metal-induced oxidative stress [33,92].

Although the 48-h exposure is operationally defined as an acute toxicity test based on the lethality endpoint (LC_50_), it represents a short-term exposure that encompasses a substantial portion of *B. plicatilis* lifespan, thereby capturing critical physiological transitions.

Overall, the metal mixture representative of scrubber washwater induced significant acute toxicity even at low concentrations, with mortality rates reaching 94% in *A. franciscana* nauplii and 71% in *B. plicatilis*. Based on the data summarized in Table 1, typical effluent metal mixture concentrations vary from approximately 15 to 32 mg/L. This comparison confirms that actual scrubber effluents contain cumulative metal concentrations that are up to 3.5 to 9.3 times higher than the thresholds required to cause 50% mortality in these marine micro-invertebrates (*A. franciscana* nauplii: 3.43 mg/L, *A. franciscana* juveniles: 10.70 mg/L, *A. franciscana* adults: 12.48 mg/L, *B. plicatilis* 4.29 mg/L; Figure 4), underscoring a severe environmental risk. These findings are consistent with previous studies reporting strong mixture effects, including significant impacts on metazooplankton at 1.5 mg/L [15] and greater-than-additive toxicity in *Acartia tonsa* [10], highlighting that co-occurring contaminants can generate synergistic effects that intensify ecological risk, particularly in filter feeders and early life stages.

### 4.3. Environmental Implications of Metal-Laden Scrubber Washwater Discharges

Heavy metal toxicity in aquatic organisms is highly species-dependent and influenced by environmental factors such as temperature, oxygen saturation, or water hardness [93]. In addition, the complexity of scrubber effluents must be considered, as they contain not only metals but also co-contaminants such as polycyclic aromatic hydrocarbons (PAHs) and suspended particulate matter [94], which can generate synergistic or antagonistic interactions not captured by simplified metal mixtures. For example, co-exposure to metals and PAHs has been associated with enhanced toxic responses due to additive effects [43]. Non-chemical stressors, including elevated temperature and dissolved organic matter (DOM), further modulate toxicity; as DOM generally changes metal bioavailability through complexation, certain conditions may destabilize these complexes and increase toxicity [95]. The ecological relevance of these complex mixtures is supported by studies showing that real scrubber washwater impairs zooplankton biodiversity, reproduction, and grazing activity [11,96] and induces abnormal larval development in invertebrates [97], with potential propagation through trophic transfer via bioaccumulation and biomagnification [10].

A particular critical factor is pH, which strongly controls metal speciation and free ion activity [98]. Fresh scrubber effluents are typically acidic, increasing the solubility and bioavailability of metals such as Fe and Zn, although speciation rapidly evolves during dilution in seawater [8]. At low pH (e.g., pH 2), metals are predominantly present as free ions or simple inorganic complexes rather than the hydroxide, carbonate, or organic forms typical of seawater (pH~8.2) [99], and these free ions are generally more bioavailable and toxic [100]. For instance, Fe(II), which dominates under acidic conditions, is more bioavailable and potentially more hazardous than Fe(III) [101], and its precipitation as ferric oxyhydroxides may also induce physical toxicity [73], which may explain the toxicity of Fe in *B. plicatilis*. Rapid pH-driven speciation changes, such as those observed between pH 6.3–6.7, can significantly alter Fe toxicity [102]. However, the relationship between pH and metal toxicity is not always predictable; modeling studies suggest increased free ion concentrations under ocean acidification [103], while experimental results show both increased Zn bioavailability at low pH [104] and reduced Zn and Cd uptake due to complexation effect [40]. The simultaneous release of multiple metals under acidic conditions may enhance synergistic toxicity, posing challenges for risk assessment frameworks that often neglect mixture interactions and speciation-dependent effects [81,105].

## 5. Conclusions

This study accurately assessed the acute toxic effects of common scrubber washwater metals (Fe, V, Ni, and Zn), individually and in mixtures, on two marine model organisms: *A. franciscana* and *B. plicatilis*. Concentration-dependent toxic effects were confirmed for all tested metals. Vanadium was consistently the most toxic metal overall, achieving LC_50_ (48 h) values below 2 mg/L in the most sensitive stages of *A. franciscana*. However, iron exhibited the lowest LC_50_ (4.89 ± 0.64 mg/L) and was thus the most toxic metal for *B. plicatilis*. Sensitivity significantly varies across species and developmental stages. Nauplii of *A. franciscana* and *B. plicatilis* were the most vulnerable organisms.

Cumulative Toxic Unit analysis of the metal mixture revealed a dynamic and life-stage-dependent toxicological impact on zooplankton. The larval stage of *A. franciscana* showed clear antagonism (TR < 1.0), suggesting strong metal competition for uptake sites. In contrast, juveniles and adults of *A. franciscana* and the rotifer *B. plicatilis* exhibited synergy (TR > 1.0), implying that the saturation of detoxification mechanisms exacerbated the overall toxicity of the mixture. These findings demonstrate that the ecotoxicological risk of scrubber metal mixtures cannot be predicted solely by simple addition and must be assessed at multiple sensitive life stages.

The comparative analysis across species and life stages reveals a fundamental ecotoxicological principle: larval stages and small zooplankton are the most sensitive bioindicators for scrubber-related contaminants. Specifically, the high vulnerability of *A. franciscana* nauplii and the rotifer *B. plicatilis* to the metal mixture and individual elements, such as Fe and V, confirms that these early life stages, which form the base of the marine food web, are at the highest ecological risk.

Overall, this work provides evidence on the potential impact of metals from scrubber washwater on marine organisms. However, metal toxicity is complex and heavily influenced by organism physiology, metal speciation, and environmental conditions; thus, generalizing toxicity predictions remains a significant challenge. Installing scrubber systems may lead to the introduction of elevated levels of heavy metals to the environment, as evidenced by their high concentrations in discharge washwater. Furthermore, this acidity modifies the mobility and chemical speciation of metals, potentially enhancing their bioavailability and subsequent ecological toxicity.

## Figures and Tables

**Table 1:** Metal concentrations found in closed-loop scrubber washwater: comparison between values reported in the previous literature and the present study.

V (µg/L)	Zn (µg/L)	Ni (µg/L)	Fe (µg/L)	Total Metal Concentration * (µg/L)	Authors
9100	370	2700	490	14,470.32	[7]
24,000	610	5700	No data	30,570.00	[22] (EGCSA dataset)
25,000	590	6600	No data	32,432.96	
14,000	420	3100	No data	18,397.40	
6100	160	3100	No data	7950.00	[29]
5641	377	1943	6620	15,573.30	This study

* Note: “Total metal concentration” refers to the cumulative sum of all trace elements analyzed in the respective effluents, including minor metals not displayed in individual columns.

**Table 2:** Observed Cumulative Toxic Unit (CTU_observed_) and Predicted Cumulative Toxic Unit (CTU_predicted_) of the metal mixture simulating a scrubber effluent in *A. franciscana* (nauplii, juveniles, and adults) and *B. plicatilis*. The CTU_observed_ was derived from the 48 h LC_50_ of the metal mixture, while the CTU_predicted_ was calculated based on the Concentration Addition (CA) model using 48 h LC_50_ values for four individual metals (V, Zn, Ni, and Fe). The Toxicity Ratio (TR = CTU_observed_/CTU_predicted_) serves as the primary tool for evaluating mixture interactions: a TR = 1.0 indicates strict additive effects; a TR < 1.0 indicates antagonistic interactions (observed toxicity is lower than predicted); and a TR > 1.0 indicates synergistic interactions (observed toxicity is higher than predicted). See complementary data in the Supplementary Material (Table S3).

Tested Organism	CTU_observed_	CTU_predicted_	TR (CTU_observed_/CTU_predicted_)	Interpretation
*A. franciscana* (nauplii)	4.37	5.75	0.76	Antagonism
*A. franciscana* (juveniles)	1.40	1.27	1.11	Synergism
*A. franciscana* (adults)	1.20	0.86	1.39	Synergism
*B. plicatilis*	3.50	2.06	1.70	Synergism

## Data Availability

The raw data supporting the conclusions of this article will be made available by the authors on request.

## Supplementary Materials

The following supporting information can be downloaded at: https://www.mdpi.com/article/10.3390/toxics14060530/s1, Figure S1: Graphical representation of the fit of the data obtained after 48 h to the model proposed by Hampel et al. (2001) [36]. (A) Nauplii of *A. franciscana* (B) Juveniles of *A. franciscana* (C) Adults of *A. franciscana* (D) *B. plicatilis.* (E) Metal mix; Table S1. Results from the stock metal analyses performed using a mass spectrometer (ICP-MS/Thermo Elemental Series-X) at the Spectroscopy Division of the “Central Science and Technology Services” of the University of Cádiz; Table S2: LC_50_, coefficient of determination (R^2^), and statistical significance (*p*-value) values corresponding to the fit of the experimental data to the sigmoid dose-response model for all organisms exposed to individual metals. The models show statistical significance when (*p* < 0.05); Table S3: Observed (CTU_observed_) and predicted (CTU_predicted_) cumulative toxic units of the metal mixture simulating a scrubber effluent in *A. franciscana* (nauplii, juveniles, adults) and *B. plicatilis*. Individual Toxic Units (TU_i_) were calculated as TU_i_ = C_i_/LC_50_, where C_i_ is the individual metal concentration in the effluent. The CTU_observed_ was derived from the mixture 48 h LC_50_, using the total mixture concentration of 15.00 mg/L (5.55 mg/L V, 6.75 mg/L Fe, 2.25 mg/L Ni, and 0.45 mg/L Zn) as the reference value (CTU_observed_ = C_mix_/LC_50mix_). The CTU_predicted_ was calculated based on the Concentration Addition (CA) model (CTU_predicted_ = ∑ TU_i_). The Toxicity Ratio (TR = CTU_observed_/CTU_predicted_) indicates additive (TR = 1.0), antagonistic (TR < 1.0), or synergistic (TR > 1.0) interactions.

## References

[1] United Nations Conference on Trade and Development (UNCTAD) (2025). Review of Maritime Transport 2025.

[2] International Maritime Organization (IMO) (1978). International Convention for the Prevention of Pollution from Ships (MARPOL).

[3] Viana M., Hammingh P., Colette A., Querol X., Degraeuwe B., de Vlieger I., van Aardenne J. (2014). Impact of maritime transport emissions on coastal air quality in Europe. Atmos. Environ..

[4] International Maritime Organization (IMO) (2019). Resolution MEPC.320(74): 2019 Guidelines for Consistent Implementation of the 0.50% Sulphur Limit Under MARPOL Annex VI.

[5] EEA-EMSA European Environment Agency & European Maritime Safety Agency (2025). European Maritime Transport Environmental Report 2025.

[6] IMO International Maritime Organization (2015). Resolution MEPC.259(68): 2015 Guidelines for Exhaust Gas Cleaning Systems.

[7] Hermansson A.L., Hassellöv I.M., Moldanova J., Ytreberg E. (2021). Comparing emissions of polyaromatic hydrocarbons and metals from marine fuels and scrubbers. Transp. Res. Part D Transp. Environ..

[8] Turner D.R., Hassellöv I.-M., Ytreberg E., Rutgersson A. (2017). Shipping and the environment: Smokestack emissions, scrubbers and unregulated oceanic consequences. Elem. Sci. Anthr..

[9] Hermansson A.L., Hassellöv I.-M., Grönholm T., Jalkanen J.-P., Fridell E., Parsmo R., Hassellöv J., Ytreberg E. (2024). Strong economic incentives of ship scrubbers promoting pollution. Nat. Sustain..

[10] Koski M., Stedmon C., Trapp S. (2017). Ecological effects of scrubber water discharge on coastal plankton: Potential synergistic effects of contaminants reduce survival and feeding of the copepod *Acartia tonsa*. Mar. Environ. Res..

[11] Jönander C., Egardt J., Hassellöv I.M., Tiselius P., Rasmussen M., Dahllöf I. (2023). Exposure to closed-loop scrubber washwater alters biodiversity, reproduction, and grazing of marine zooplankton. Front. Mar. Sci..

[12] Ytreberg E., Hassellöv I.M., Nylund A.T., Hedblom M., Al-Handal A.Y., Wulff A. (2019). Effects of scrubber washwater discharge on microplankton in the Baltic Sea. Mar. Pollut. Bull..

[13] Genitsaris S., Kourkoutmani P., Stefanidou N., Michaloudi E., Gros M., García-Gómez E., Petrović M., Ntziachristos L., Moustaka-Gouni M. (2023). Effects from maritime scrubber effluent on phytoplankton and bacterioplankton communities of a coastal area, Eastern Mediterranean Sea. Ecol. Inform..

[14] Endres S., Maes F., Hopkins F., Houghton K., Mårtensson E.M., Oeffner J., Quack B., Singh P., Turner D. (2018). A new perspective at the ship-air-sea-interface: The environmental impacts of exhaust gas scrubber discharge. Front. Mar. Sci..

[15] Kourkoutmani P., Genitsaris S., Demertzioglou M., Stefanidou N., Voutsa D., Ntziachristos L., Moustaka-Gouni M., Michaloudi E. (2024). Effects from maritime scrubber effluent on coastal metazooplankton. Mar. Biol..

[16] Zapata-Restrepo L.M., Williams I.D., Hudson M., Freeman G., Lee B., Prieul C. (2024). Ecotoxicological assessment of waste scrubber water in unicellular algae (*Tetraselmis suecica*) and Blue Mussel (*Mytilus edulis*) larvae. Detritus.

[17] El-Sharkawy M., Alotaibi M.O., Li J., Du D., Mahmoud E. (2025). Heavy Metal Pollution in Coastal Environments: Ecological Implications and Management Strategies: A Review. Sustainability.

[18] Väänänen K., Leppänen M.T., Chen X., Akkanen J. (2018). Metal bioavailability in ecological risk assessment of freshwater ecosystems: From science to environmental management. Ecotoxicol. Environ. Saf..

[19] Nys C., Versieren L., Cordery K.I., Blust R., Smolders E., De Schamphelaere K.A. (2017). Systematic evaluation of chronic metal-mixture toxicity to three species and implications for risk assessment. Environ. Sci. Technol..

[20] Gleyzes C., Tellier S., Astruc M. (2002). Fractionation studies of trace elements in contaminated soils and sediments: A review of sequential extraction procedures. TrAC Trends Anal. Chem..

[21] Sarkar S.K., Favas P.J., Rakshit D., Satpathy K.K. (2014). Geochemical speciation and risk assessment of heavy metals in soils and sediments. Environmental Risk Assessment of Soil Contamination.

[22] Teuchies J., Cox T.J.S., Van Itterbeeck K., Meysman F.J.R., Blust R. (2020). The impact of scrubber discharge on the water quality in estuaries and ports. Environ. Sci. Eur..

[23] Norwood W.P., Borgmann U., Dixon D.G., Wallace A. (2003). Effects of Metal Mixtures on Aquatic Biota: A Review of Observations and Methods. Hum. Ecol. Risk Assess. Int. J..

[24] Rainbow P.S. (2007). Trace metal bioaccumulation: Models, metabolic availability and toxicity. Environ. Int..

[25] Kukavica B., Davidović-Plavšić B., Savić A., Dmitrović D., Šukalo G., Đurić-Savić S., Vučić G. (2023). Oxidative stress and neurotoxicity of cadmium and zinc on *Artemia franciscana*. Biol. Trace Elem. Res..

[26] Enserink E.L., Mass-Diepeveen J.L., van Leeuwen C.J. (1991). Combined effects of metals: An ecotoxicological evaluation. Water Res..

[27] Knežević L., Omanović D., Bačić N., Mandić J., Bura-Nakić E. (2021). Redox Speciation of Vanadium in Estuarine Waters Using Improved Methodology Based on Anion Exchange Chromatography Coupled to HR ICP-MS System. Molecules.

[28] Kremling K., Andreae M.O., Brügmann L., van den Berg C.M.G., Prange A., Schirmacher M., Koroleff E., Kus J., Grasshoff K., Kremling K., Ehrhardt M. (1999). Determination of trace elements. Methods of Seawater Analysis.

[29] Kjølholt J., Aakre S., Jürgensen C., Lauridsen J. (2012). Assessment of Possible Impacts of Scrubber Water Discharges on the Marine Environment.

[30] Libralato G., Prato E., Migliore L., Cicero A.M., Manfra L. (2016). A review of toxicity testing protocols and endpoints with *Artemia* spp. Ecol. Indic..

[31] Li X.D., Wang X.Y., Xu M.E., Jiang Y., Yan T., Wang X.C. (2020). Progress on the usage of the rotifer *Brachionus plicatilis* in marine ecotoxicology: A review. Aquat. Toxicol..

[32] Van Steertegem M., Persoone G., Soares A.M.V.M., Calow P. (1993). Cyst-based toxicity tests V: Development and critical evaluation of standardized toxicity tests with the brine shrimp Artemia (anostraca, Crustacea). Progress in Standardization of Aquatic Toxicity Tests.

[33] Snell T.W., Janssen C.R. (1995). Rotifers in ecotoxicology: A review. Hydrobiologia.

[34] Serra M., Carmona M.J., Miracle M.R. (1994). Survival analysis of three clones of *Brachionus plicatilis* (Rotifera). Hydrobiologia.

[35] Yin X.W., Zhao W. (2008). Studies on life history characteristics of *Brachionus plicatilis* OF Müller (Rotifera) in relation to temperature, salinity and food algae. Aquat. Ecol..

[36] Hampel M., Moreno-Garrido I., Sobrino C., Lubián L.M., Blasco J. (2001). Acute Toxicity of LAS Homologues in Marine Microalgae: Esterase Activity and Inhibition Growth as Endpoints of Toxicity. Ecotoxicol. Environ. Saf..

[37] Altenburger R., Nendza M., Schüürmann G. (2003). Mixture toxicity and its modeling by quantitative structure-activity relationships. Environ. Toxicol. Chem..

[38] Backhaus T., Faust M. (2012). Predictive environmental risk assessment of chemical mixtures: A conceptual framework. Environ. Sci. Technol..

[39] Sprague J.B. (1970). Measurement of pollutant toxicity to fish. II. Utilizing and applying bioassay results. Water Res..

[40] Xu Y., Shi D., Aristilde L., Morel F.M. (2012). The effect of pH on the uptake of zinc and cadmium in marine phytoplankton: Possible role of weak complexes. Limnol. Oceanogr..

[41] Martin O., Scholze M., Ermler S., McPhie J., Bopp S.K., Kienzler A., Parissis N., Kortenkamp A. (2021). Ten years of research on synergisms and antagonisms in chemical mixtures: A systematic review and quantitative reappraisal of mixture studies. Environ. Int..

[42] Kumar P., Sivaperumal P., Manigandan V., Rajaram R., Hussain M. (2021). Assessment of potential human health risk due to heavy metal contamination in edible finfish and shellfish collected around Ennore coast, India. Environ. Sci. Pollut. Res..

[43] Gauthier P.T., Norwood W.P., Prepas E.E., Pyle G.G. (2014). Metal–PAH mixtures in the aquatic environment: A review of co-toxic mechanisms leading to more-than-additive outcomes. Aquat. Toxicol..

[44] Valko M.M.H.C.M., Morris H., Cronin M.T.D. (2005). Metals, toxicity and oxidative stress. Curr. Med. Chem..

[45] Meina E.G., Niyogi S., Liber K. (2020). Investigating the mechanism of vanadium toxicity in freshwater organisms. Aquat. Toxicol..

[46] Fichet D., Miramand P. (1998). Vanadium toxicity to three marine invertebrates larvae: *Crassostrea gigas*, *Paracentrotus lividus* and *Artemia salina*. Chemosphere.

[47] Tambat V.S., Patel A.K., Singhania R.R., Chen C.-W., Dong C.-D. (2024). Marine vanadium pollution: Sources, ecological impacts and cutting-edge mitigation strategies. Mar. Pollut. Bull..

[48] Chiarelli R., Martino C., Roccheri M.C., Geraci F. (2022). Vanadium Toxicity Monitored by Fertilization Outcomes and Metal-Related Proteolytic Activities in *Paracentrotus lividus* Embryos. Toxics.

[49] Hobbs A.S., Froehlich J.P., Albers R.W. (1980). Inhibition by vanadate of the reactions catalyzed by the (Na++ K+)-stimulated ATPase. A transient state kinetic characterization. J. Biol. Chem..

[50] Bell M.V., Sargent J.R. (1979). The partial purification of sodium-plus-potassium ion-dependent adenosine triphosphatase from the gills of Anguilla anguilla and its inhibition by orthovanadate. Biochem. J..

[51] Ringelband U. (2001). Salinity dependence of vanadium toxicity against the brackish water hydroid *Cordylophora caspia*. Ecotoxicol. Environ. Saf..

[52] Sellami I., Charmantier G., Naceur H.B., Kacem A., Lorin-Nebel C. (2020). Osmoregulatory performance and immunolocalization of Na^+^/K^+^-ATPase in the branchiopod *Artemia salina* from the Sebkha of Sidi El Hani (Tunisia). Tissue Cell.

[53] El-Agawany N.I., Kaamoush M.I. (2023). Role of zinc as an essential microelement for algal growth and concerns about its potential environmental risks. Environ. Sci. Pollut. Res..

[54] Fisher N.S., Hook S.E. (2002). Toxicology tests with aquatic animals need to consider the trophic transfer of metals. Toxicology.

[55] Hazeem L. (2022). Single and combined toxicity effects of zinc oxide nanoparticles: Uptake and accumulation in marine microalgae, toxicity mechanisms, and their fate in the marine environment. Water.

[56] Marisa I., Matozzo V., Munari M., Binelli A., Parolini M., Martucci A., Franceschinis E., Brianese N., Marin M.G. (2016). In vivo exposure of the marine clam *Ruditapes philippinarum* to zinc oxide nanoparticles: Responses in gills, digestive gland and haemolymph. Environ. Sci. Pollut. Res..

[57] Muyssen B.T., De Schamphelaere K.A., Janssen C.R. (2006). Mechanisms of chronic waterborne Zn toxicity in Daphnia magna. Aquat. Toxicol..

[58] Wang W.X., Ke C. (2002). Dominance of dietary intake of cadmium and zinc by two marine predatory gastropods. Aquat. Toxicol..

[59] Anu P.R., Nandan S.B., Jayachandran P.R., Xavier N.D., Midhun A.M., Mohan D. (2018). Toxicity effects of zinc on two marine diatoms, under varying macronutrient environment. Mar. Environ. Res..

[60] Mohammadi S., Ahmadifard N., Atashbar B., Nikoo A., Manaffar R. (2021). Long-term effect of zinc oxide nanoparticles on population growth, reproductive characteristics and zinc accumulation of marine rotifer, *Brachionus plicatilis*. Int. J. Aquat. Biol..

[61] Blewett T.A., Leonard E.M. (2017). Mechanisms of nickel toxicity to fish and invertebrates in marine and estuarine waters. Environ. Pollut..

[62] Brix K.V., Schlekat C.E., Garman E.R. (2017). The mechanisms of nickel toxicity in aquatic environments: An adverse outcome pathway analysis. Environ. Toxicol. Chem..

[63] Magyarosy A., Laidlaw R., Kilaas R., Echer C., Clark D., Keasling J. (2002). Nickel accumulation and nickel oxalate precipitation by *Aspergillus niger*. Appl. Microbiol. Biotechnol..

[64] Binet M.T., Adams M.S., Gissi F., Golding L.A., Schlekat C.E., Garman E.R., Merrington G., Stauber J.L. (2018). Toxicity of nickel to tropical freshwater and sediment biota: A critical literature review and gap analysis. Environ. Toxicol. Chem..

[65] Deruytter D., Baert J.M., Nevejan N., De Schamphelaere K.A., Janssen C.R. (2017). Mixture toxicity in the marine environment: Model development and evidence for synergism at environmental concentrations. Environ. Toxicol. Chem..

[66] Martin M., Osborn K.E., Billig P., Glickstein N. (1981). Toxicities of ten metals to Crassostrea gigas and *Mytilus edulis* embryos and Cancer magister larvae. Mar. Pollut. Bull..

[67] Rebolledo U.A., Páez-Osuna F., Fernández R. (2021). Single and mixture toxicity of As, Cd, Cr, Cu, Fe, Hg, Ni, Pb, and Zn to the rotifer *Proales similis* under different salinities. Environ. Pollut..

[68] Migliore L., de Nicola Giudici M. (1990). Toxicity of heavy metals to *Asellus aquaticus* (L.) (Crustacea, Isopoda). Hydrobiologia.

[69] Langston W.J., Furness R.W., Rainbow P.S. (1990). Toxic Effects of Heavy Metals and the Incidence of Metal Pollution in Marine Ecosystems. Heavy Metals in the Marine Environment.

[70] Nakamura T., Naguro I., Ichijo H. (2019). Iron homeostasis and iron-regulated ROS in cell death, senescence and human diseases. Biochim. Biophys. Acta.

[71] Wang H., Tang X., Sha J., Chen H., Sun T., Wang Y. (2015). The reproductive toxicity on the rotifer *Brachionus plicatilis* induced by BDE-47 and studies on the effective mechanism based on antioxidant defense system changes. Chemosphere.

[72] Han C., Kim H.-J., Lee J.-S., Sakakura Y., Hagiwara A. (2022). Iron reproductive toxicity of marine rotifer sibling species: Adaptation to temperate and tropical habitats. Aquat. Toxicol..

[73] Couillard Y., Ross P., Pinel-Alloul B. (1989). Acute toxicity of six metals to the Rotifer *Brachionus calyciflorus*, with comparisons to other freshwater organisms. Toxic. Assess..

[74] Jansen M., Groman D. (1993). The Effect of High Concentrations of Iron on Impounded American Lobsters: A Case Study. J. Aquat. Anim. Health.

[75] MacRae T.H., Pandey A.S. (1991). Effects of metals on early life stages of the brine shrimp, Artemia: A developmental toxicity assay. Arch. Environ. Contam. Toxicol..

[76] Gora A.H., Sreeram M.P., Rehman S., Ain Q.U., Chakraborty K., Prema D., Lavanya R., Siriyappagouder P., Asha P.S. (2025). A review on metallothionein research in marine and estuarine realms: Past paradigms and future vistas. Front. Mar. Sci..

[77] Manzo S., Buono S., Cremisini C. (2010). Cadmium, lead and their mixtures with copper: Paracentrotus lividus embryotoxicity assessment, prediction, and offspring quality evaluation. Ecotoxicology.

[78] Koppel D.J., Adams M.S., King C.K., Jolley D.F. (2018). Chronic toxicity of an environmentally relevant and equitoxic ratio of five metals to two Antarctic marine microalgae shows complex mixture interactivity. Environ. Pollut..

[79] Crowther C., Turner A., Moore M.N., Jha A.N. (2023). Assessing the effects of single and binary exposures of copper and lead on *Mytilus galloprovincialis*: Physiological and genotoxic approaches. Aquat. Toxicol..

[80] Liu F., Wang W.X. (2014). Differential influences of Cu and Zn chronic exposure on Cd and Hg bioaccumulation in an estuarine oyster. Aquat. Toxicol..

[81] Xu X., Li Y., Wang Y., Wang Y. (2011). Assessment of toxic interactions of heavy metals in multi-component mixtures using sea urchin embryo-larval bioassay. Toxicol. Vitr..

[82] Amachree D., Moody A.J., Handy R.D. (2024). Bioaccumulation and sub-lethal physiological effects of metal mixtures on mussel, *Mytilus edulis*: Continuous exposure to a binary mixture of mercury and cadmium. Aquat. Toxicol..

[83] Di Toro D.M., Allen H.E., Bergman H.L., Meyer J.S., Paquin P.R., Santore R.C. (2001). Biotic Ligand Model of the acute toxicity of metals. 1. Technical basis. Environ. Toxicol. Chem..

[84] Niyogi S., Wood C.M. (2004). Biotic ligand model, a flexible tool for developing site-specific water quality guidelines for metals. Environ. Sci. Technol..

[85] Sun D.Y., Guo J.Z., Hartmann H.A., Uno H.I.D.E.O., Hokin L.E. (1991). Na, K-ATPase expression in the developing brine shrimp Artemia. Immunochemical localization of the alpha-and beta-subunits. J. Histochem. Cytochem..

[86] Griffith M.B. (2017). Toxicological perspective on the osmoregulation and ionoregulation physiology of major ions by freshwater animals: Teleost fish, crustacea, aquatic insects, and Mollusca. Environ. Toxicol. Chem..

[87] Peace A., Poteat M.D., Wang H. (2016). Somatic growth dilution of a toxicant in a predator–prey model under stoichiometric constraints. J. Theor. Biol..

[88] Amiard J.-C., Amiard-Triquet C., Barka S., Pellerin J., Rainbow P.S. (2006). Metallothioneins in aquatic invertebrates: Their role in metal detoxification and their use as biomarkers. Aquat. Toxicol..

[89] Ivanina A.V., Cherkasov A.S., Sokolova I.M. (2008). Effects of cadmium on cellular protein and glutathione synthesis and expression of stress proteins in eastern oysters, Crassostrea virginica Gmelin. J. Exp. Biol..

[90] Gambardella C., Mesarič T., Milivojević T., Sepčić K., Gallus L., Carbone S., Ferrando S., Faimali M. (2014). Effects of selected metal oxide nanoparticles on Artemia salina larvae: Evaluation of mortality and behavioural and biochemical responses. Environ. Monit. Assess..

[91] Li Y., Chai X., Wu H., Jing W., Wang L. (2013). The response of metallothionein and malondialdehyde after exclusive and combined Cd/Zn exposure in the crab *Sinopotamon henanense*. PLoS ONE.

[92] Rico-Martínez R., Arzate-Cárdenas M.A., Robles-Vargas D., Pérez-Legaspi I.A., Alvarado-Flores J., Santos-Medrano G.E. (2016). Rotifers as Models in Toxicity Screening of Chemicals and Environmental Samples. Invertebrates Experimental Models in Toxicity Screening.

[93] Santhosh K., Kamala K., Ramasamy P., Musthafa M.S., Almujri S.S., Asdaq S.M.B., Sivaperumal P. (2024). Unveiling the silent threat: Heavy metal toxicity devastating impact on aquatic organisms and DNA damage. Mar. Pollut. Bull..

[94] Gondikas A., Mattsson K., Hassellöv M. (2025). A new form of hazardous microparticulate contamination to the marine environment from ships using heavy fuel oil with exhaust gas scrubbers—Characterization and implications for fate, transport and ecotoxicity. Sci. Total Environ..

[95] Doig L.E., Liber K. (2006). Influence of dissolved organic matter on nickel bioavailability and toxicity to Hyalella azteca in water-only exposures. Aquat. Toxicol..

[96] Thor P., Granberg M.E., Winnes H., Magnusson K. (2021). Severe toxic effects on pelagic copepods from maritime exhaust gas scrubber effluents. Environ. Sci. Technol..

[97] Zapata-Restrepo L.M., Williams I.D. (2025). Mytilus edulis and *Psammechinus miliaris* as bioindicators of ecotoxicological risk by maritime exhaust gas scrubber water. Mar. Environ. Res..

[98] Picone M., Russo M., Distefano G.G., Baccichet M., Marchetto D., Volpi Ghirardini A., Lunde Hermansson A., Petrovic M., Gros M., Garcia E. (2023). Impacts of exhaust gas cleaning systems (EGCS) discharge waters on planktonic biological indicators. Mar. Pollut. Bull..

[99] Byrne R.H. (2003). Inorganic speciation of dissolved elements in seawater: The influence of pH on concentration ratios. Geochem. Trans..

[100] Millero F. (2001). Speciation of metals in natural waters. Geochem. Trans..

[101] Cardwell A.S., Rodriguez P.H., Stubblefield W.A., DeForest D.K., Adams W.J. (2023). Chronic toxicity of iron to aquatic organisms under variable pH, hardness, and dissolved organic carbon conditions. Environ. Toxicol. Chem..

[102] Teien H.C., Garmo A.C., Atland A., Salbu B. (2008). Transformation of iron species in mixing zones and accumulation on fish gills. Environ. Sci. Technol..

[103] Stockdale A., Tipping E., Lofts S., Mortimer R.J.G. (2016). The effect of ocean acidification on organic and inorganic speciation of trace metals. Environ. Sci. Technol..

[104] De Orte M.R., Lombardi A.T., Sarmiento A.M., Basallote M.D., Rodríguez-Romero A., Riba I., Del Valls A. (2014). Metal mobility and toxicity to microalgae associated with acidification of sediments: CO_2_ and acid comparison. Mar. Environ. Res..

[105] Shah S.B. (2021). Heavy Metals in the Marine Environment—An Overview. Heavy Metals in Scleractinian Corals.

