# Supplementary material for: Life Stage-Dependent Toxicity and Interactions of Scrubber-Related Metal Mixtures in Marine Zooplankton
Source: Toxics. 2026 Jun 19;14(6):530. doi: 10.3390/toxics14060530 (PMC13307446; doi:10.3390/toxics14060530)
Supplement: Supplementary file 1 [file toxics-14-00530-s001.zip › toxics-4272841-supplementary.pdf]

toxics

MDPI

Supplementary Materials

Life Stage-Dependent Toxicity and Interactions of Scrubber-Related Metal Mixtures in Marine Zooplankton

Esther Bautista-Chamizo [1,2,*], María Cabrera-Bayarri [1], Enrique Nebot [1] and Javier Moreno-Andrés [1]

[1] Department of Environmental Technologies, Marine Research Institute (INMAR), Faculty of Marine and Environmental Sciences, University of Cádiz, 11510 Puerto Real, Spain; maria.cabrerabayarri@alum.uca.es (M.C.-B.); enrique.nebot@uca.es (E.N.); javier.moreno@uca.es (J.M.-A.)
[2] Microbiology and Proteomics Laboratory, Department of Biomedicine, Biotechnology and Public Health, Institute for Viticulture and Agri-Food Research (IVAGRO), Faculty of Marine and Environmental Sciences, University of Cádiz, 11510 Puerto Real, Spain
* Correspondence: esther.bautista@uca.es

Academic Editor: Xiaoshan Zhu

Received: 6 April 2026
Revised: 26 May 2026
Accepted: 11 June 2026
Published: 19 June 2026

Copyright: © 2026 by the authors. Licensee MDPI, Basel, Switzerland. This article is an open access article distributed under the terms and conditions of the Creative Commons Attribution (CC BY) license.

 https://doi.org/10.3390/toxics14060530

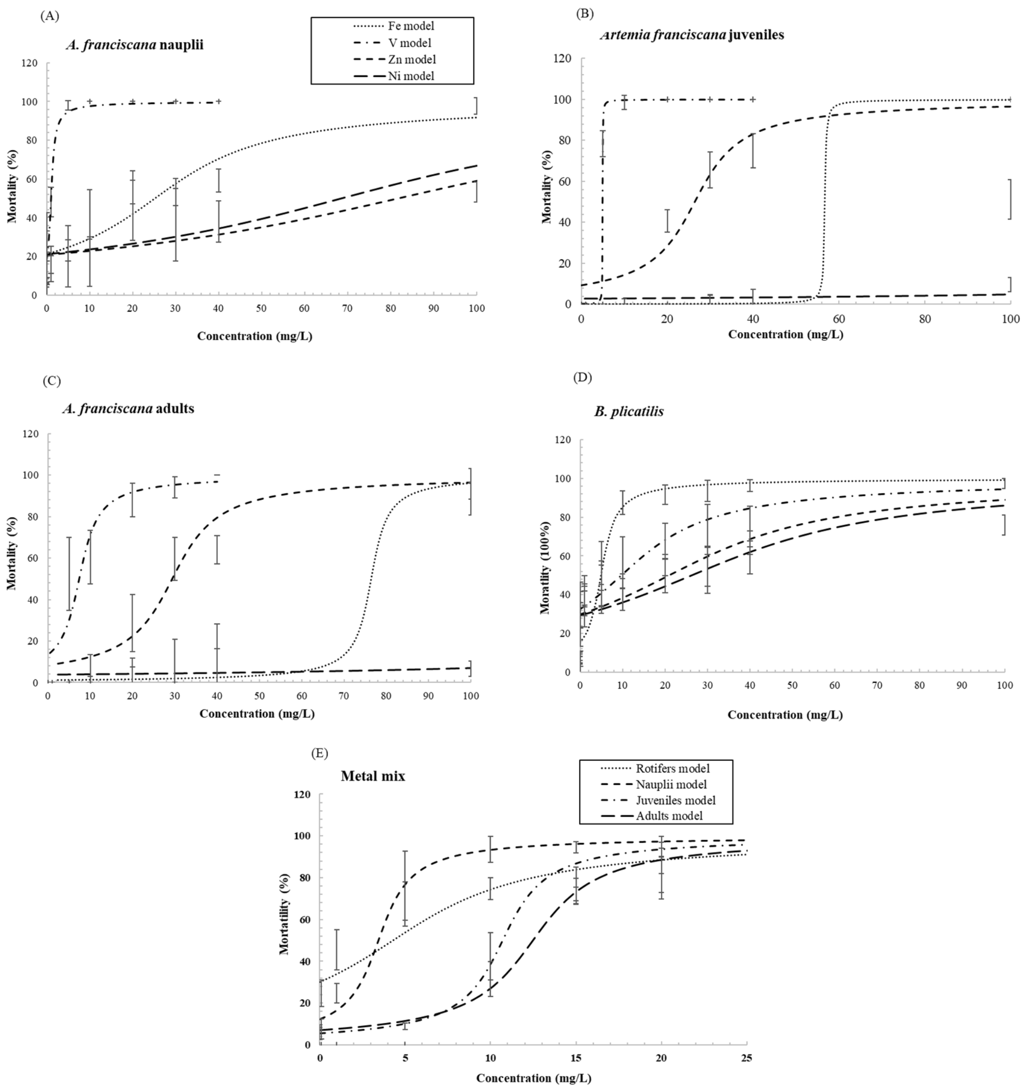

Figure S1. Graphical representation of the fit of the data obtained after 48 hours to the model proposed by Hampel et al. (2001) [36]. A. Nauplii of *A. franciscana* B. Juveniles of *A. franciscana* C. Adults of *A. franciscana* D. *B. plicatilis.* E. Metal mix.

Table S1. Results from the stock metal analyses performed using a mass spectrometer (ICP-MS / Thermo Elemental Series-X) at the Spectroscopy Division of the "Central Science and Technology Services" of the University of Cádiz.

	Concentration	
	Nominal	Measured
V stock	100 mg/L	100 ± 2
Zn stock	250 mg/L	251 ± 4
Ni stock	100 mg/L	99 ± 1
Fe stock	250 mg/L	253 ± 5
Filtered Fe stock	250 mg/L	199 ± 1

Table S2. LC_{50}, coefficient of determination (R^2), and statistical significance (*p*-value) values corresponding to the fit of the experimental data to the sigmoid dose-response model for all organisms exposed to individual metals. The models show statistical significance when ($p < 0.05$).

Organism	Metal	LC_{50}	R^2	*p*-Value
A. franciscana (nauplii)	Vanadium	1.02	0.97	0.0005
	Zinc	81.79	0.87	0.0002
	Nickel	68.59	0.87	0.0003
	Iron	25.18	0.93	<0.0001
A. franciscana (juveniles)	Vanadium	4.95	0.99	<0.0001
	Zinc	24.71	0.91	<0.0001
	Nickel	231.32	0.73	<0.0001
	Iron	56.69	0.99	0.0446
A. franciscana (adults)	Vanadium	7.38	0.91	0.0036
	Zinc	29.37	0.94	<0.0001
	Nickel	216.32	0.84	<0.0001
	Iron	76.39	0.99	<0.0001
B. plicatilis	Vanadium	9.68	0.84	0.0135
	Zinc	20.93	0.91	0.0008
	Nickel	26.25	0.89	0.0009
	Iron	4.89	0.95	<0.0001

Table S3. Observed ($CTU_{observed}$) and predicted ($CTU_{predicted}$) cumulative toxic units of the metal mixture simulating a scrubber effluent in *A. franciscana* (nauplii, juveniles, adults) and *B. plicatilis*. Individual Toxic Units (TU_i) were calculated as $TU_i = C_i / LC_{50}$, where C_i is the individual metal concentration in the effluent. The $CTU_{observed}$ was derived from the mixture 48 h LC_{50}, using the total mixture concentration of 15.00 mg/L (5.55 mg/L V, 6.75 mg/L Fe, 2.25 mg/L Ni, and 0.45 mg/L Zn) as the reference value ($CTU_{observed} = C_{mix} / LC_{50mix}$). The $CTU_{predicted}$ was calculated based on the Concentration Addition (CA) model ($CTU_{predicted} = \sum TU_i$). The Toxicity Ratio ($TR = CTU_{observed} / CTU_{predicted}$) indicates additive (TR = 1.0), antagonistic (TR < 1.0), or synergistic (TR > 1.0) interactions.

***A. franciscana* nauplii**	**C_i in effluent (mg/L)**	**LC_{50i} (mg/L)**	**TU_i (C_i/LC_{50i}) / CTU**
**Vanadium**	5.55	1.02	5.445
**Zinc**	0.45	81.79	0.006
**Nickel**	2.25	68.59	0.033
**Iron**	6.75	25.18	0.268
**$CTU_{predicted}$**			5.752
**$CTU_{observed}$ (15 mg/L)**		3.43	4.367

***A. franciscana* juveniles**	**C_i in effluent (mg/L)**	**LC_{50i} (mg/L)**	**$TU_i = C_i/LC_{50i}$**
**Vanadium**	5.55	4.95	1.122
**Zinc**	0.45	24.71	0.018
**Nickel**	2.25	231.32	0.010
**Iron**	6.75	56.69	0.119
**$CTU_{predicted}$**			1.269
**$CTU_{observed}$ (15 mg/L)**		10.70	1.401

***A. franciscana* adults**	**C_i in effluent (mg/L)**	**LC_{50i} (mg/L)**	**$TU_i = C_i/LC_{50i}$**
**Vanadium**	5.55	7.39	0.751
**Zinc**	0.45	29.37	0.015
**Nickel**	2.25	216.32	0.010
**Iron**	6.75	76.39	0.088
**$CTU_{predicted}$**			0.865
**$CTU_{observed}$ (15 mg/L)**		12.48	1.202

B. plicatilis	**C_i in effluent (mg/L)**	**LC_{50i} (mg/L)**	**$TU_i = C_i/LC_{50i}$**
**Vanadium**	5.55	9.68	0.573
**Zinc**	0.45	20.93	0.022
**Nickel**	2.25	26.25	0.086
**Iron**	6.75	4.89	1.380
**$CTU_{predicted}$**			2.060
**$CTU_{observed}$ (15 mg/L)**		4.29	3.499

Disclaimer/Publisher's Note: The statements, opinions and data contained in all publications are solely those of the individual author(s) and contributor(s) and not of MDPI and/or the editor(s). MDPI and/or the editor(s) disclaim responsibility for any injury to people or property resulting from any ideas, methods, instructions or products referred to in the content.

https://doi.org/10.3390/toxics14060530
